# Case Report: Neurologic signs following rattlesnake envenomation

**DOI:** 10.3389/fvets.2026.1756610

**Published:** 2026-04-07

**Authors:** Laura Weintraub, C. Langdon Fielding, Jessica Bouton, Diane Rhodes

**Affiliations:** Loomis Basin Equine Medical Center, Penryn, PA, United States

**Keywords:** bladder, neurologic, snakebite, urinary, venom

## Abstract

Rattlesnake envenomation is a common emergency in horses in California. Rattlesnakes belong to a group of venomous snakes: pit vipers. The Northern Pacific Rattlesnake (*Crotalus oreganus oreganus*), a subspecies of the Western Rattlesnake, is the most widespread rattlesnake in California (1). A 5-year-old miniature horse mare presented for a presumed Western Rattlesnake bite. The owners found the horse the morning of presentation with severe facial swelling and the horse was housed in an area where rattlesnakes are commonly observed. The horse was reported to be normal the day prior to presentation. On blood examination, severe thrombocytopenia, elevated AST, and elevated creatine kinase (CK) were identified. On physical examination the horse was noted to have severe facial swelling, to be dribbling urine, and to have a hypermetric gate in the hind limbs. Treatment was initiated with antivenom, bethanechol, and supportive care. After 6 days of hospitalization, the horse regained urinary function, the facial swelling significantly improved, the neurologic gait resolved, and the horse was discharged. This is the first case report describing neurologic manifestations from a presumed Western Rattlesnake bite.

## Introduction

Snake venoms have a diverse mixture of molecules that can lead to hemotoxic, cytotoxic, cardiotoxic, myotoxic, and neurotoxic effects. *Crotalus scutulatus*, commonly known as the Mojave rattlesnake, is well known for its venom having neurotoxic effects; however, other pit viper venoms can have certain neurotoxic components as well. Rattlesnake venom consists of a variety of compounds including proteases, phospholipases, and metalloproteases. Viper venom is mostly comprised of larger molecules that are taken up slowly through the lymphatics, which usually results in severe local effects (2). However, the clinical effect of the snakebites can range from mild local reactions to life-threatening complications. Common hemotoxic signs in humans include intense pain, edema, swelling, numbness or tingling, weakness, ecchymoses, muscle fasciculation, paresthesia, an unusual metallic taste, vomiting, confusion, and bleeding disorders (3). Local swelling at the site of the bite becomes apparent quickly and can be marked and progress over 2–3 days (2). In severe cases, systemic manifestations include acute renal failure, hypovolemic shock, disseminated intravascular coagulation, and death (2). In humans, children are more likely to suffer significant morbidity and mortality because they receive a larger envenomation relative to body size (4).

## Case description

**Figure 1:**
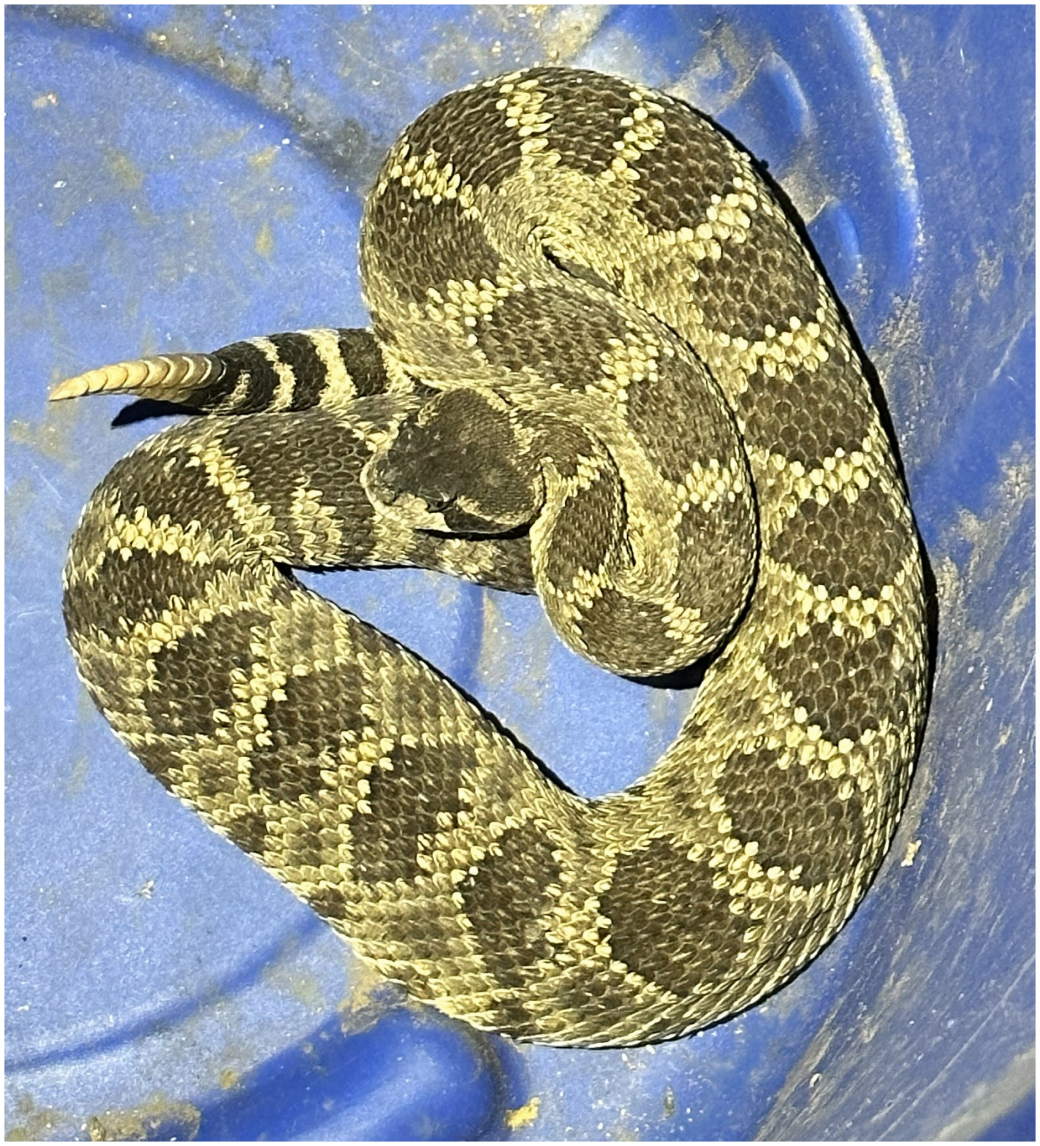
A western rattlesnake.

A 5-year-old miniature horse mare presented for a presumed Western Rattlesnake bite on the muzzle (Figure 1). No treatments were initiated prior to arrival at the hospital. The bite was not observed, and the precise interval between the inciting event and presentation is unknown. The horse was reportedly clinically normal the night prior to presentation. Upon admission, the horse had progressive, severe facial swelling and was dribbling urine. Fang marks between the nostrils were observed. The patient was noted to be hypermetric symmetrically in both hind legs. No other neurological deficits were recorded. Initial examination revealed tachycardia (68 beats per minute; RI 36–44 bpm), tachypnea (28 breaths per minute; RI 8–16 brpm), and dyspnea. A tracheostomy was attempted due to upper airway obstruction from severe facial swelling. The procedure was unsuccessful due to the fractious and uncooperative nature of the patient. The patient was then sedated with 0.02 mg/kg detomidine intramuscularly to facilitate further handling. Upper airway patency was maintained with the placement of nasal tubes. The nasal tubes were made of 10cm-cut 38 French nasogastric tubes that were secured to the halter with umbilical tape placed through the side holes.

Complete blood count revealed a mild anemia with a hematocrit (HCT) of 24.8% (reference range 30%−47%), mild neutrophilia of 8.83 × 10^9^/L (reference range 2.5–6.9 × 10^9^/L), mild lymphopenia of 1.40 × 10^9^/L (reference range 1.50–5.10 × 10^9^/L), and severe thrombocytopenia of 0 × 10^9^/L (reference range 100–250 × 10^9^/L). The hospital typically confirms that platelet counts are low with a blood smear but an exact count from a blood smear was not recorded in the medical record. Serum chemistry revealed hyperglycemia of 180 mg/dL (reference 64–150 mg/dL), hypocalcemia of 8.5 mg/dL (reference range 10.4–12.9 mg/dL), unreadable AST (reference range 100–600 U/L), and elevated CK of 1,529 U/L (reference range 10–350 U/L). Prothrombin time was 18.3 s (reference range 16–20 s) and PTT was elevated at >200 s (reference 35.8–48.6 s). Mild peripheral hyperlactatemia was 1.5 mmol/L (reference range 0–1.4 mmol/L).

The medical record noted that the administration of intravenous and intramuscular injections resulted in hematoma formation and persistent bleeding. Placement of the IV catheter also resulted in hematoma formation. The hematomas formed from the catheter and injection sites were managed with compression wraps.

An ultrasound of the bladder revealed a large bladder of approximately 12 cm. The wall was 2 mm thick and turgid. There was anechoic fluid within the bladder. Free fluid was not identified within the abdomen.

A 14-gauge polyurethane intravenous catheter was placed in the left jugular vein. Two vials of Boehringer Ingelheim Crotalidae Polyvalent Antivenin were administered, diluted in 250 mls of 0.9% sodium chloride. A vial was administered initially on presentation. After admission into the hospital, the facial swelling worsened and the horse continued to develop hematomas at venipuncture sites. A second vial of antivenom was given later on the first day of hospitalization due to the progressing clinical signs.

Urinary retention was managed by placement of an indwelling urinary catheter. After placement of the urinary catheter, copious amounts of cloudy urine was obtained. No further diagnostics were performed on the urine obtained. The horse was challenged daily via blockage of the urinary catheter. The urinary catheter remained in place for a total of 6 days. Bethanechol (0.3 mg/kg PO q8h) was given on day 6 to promote normal urination. After two doses of bethanechol, the patient was challenged again, successfully able to urinate, and the urinary catheter was removed.

Intravenous fluid therapy with Norm R was administered at 2 mls/kg/h for supportive care and to prevent acute kidney injury associated with rhabdomyolysis and the rattlesnake bite. A solution of 50% dextrose was administered at 0.4 mg/kg/min to prevent hyperlipidemia. For pain management, the horse received flunixin meglumine 1.1 mg/kg IV q12h and lidocaine (0.05 mg/kg/min) CRI. Omeprazole (1 mg/kg PO q24h) was initiated for gastric ulcer prophylaxis. Antibiotics were administered for the rattlesnake bite and due to the indwelling urinary catheter. Trimethoprim sulfamethoxazole (30 mg/kg BWT PO q12) was given during the course of hospitalization. Gentamicin (6.6 mg/kg IV) was administered once at presentation. A tetanus toxoid was given IM for tetanus prophylaxis.

Additional treatments included neomycin, polymyxin, and bacitracin ophthalmic ointment applied to both eyes twice per day due to ocular swelling. Soupy mashes and small handfuls of hay were offered frequently. As part of hospital protocol, a fecal sample was submitted for salmonella PCR testing, which was negative.

A repeat CBC was performed 2 days after admission and revealed improvement in thrombocytopenia 99 x 10^9^/L (reference range 100–250 × 10^9^/L) and anemia (HCT 22.3%; reference range 30%−47%). The hypermetric gait resolved after 48 h. The facial swelling significantly improved by the time of discharge and the excessive bleeding resolved. After 6 days of hospitalization, the patient made a full recovery and was discharged. No recurrence of urinary signs were reported. Written consent was obtained from the owners for inclusion of their animal's case information in this study.

## Comments

This is the first case report describing neurologic signs after presumed Western Rattlesnake envenomation. Given the presence of fang marks, other concurrent clinical signs, and geographic inference, a snake bite was considered the most likely etiology. Domestic animal snake bite envenomation is a common occurrence in the United States, with estimates of 150,000–200,000 occurrences per year in dogs and cats (5). Most of these are due to bites from pit vipers. Common effects reported in equids include swelling, local pain, ecchymosis, tissue necrosis, coagulopathy, and increased vascular permeability. Less frequently observed effects are cardiac arrhythmias, central nervous system dysfunction, acute renal failure, respiratory distress, hypotension, and death (5).

In a retrospective study of horses, clinical signs included head swelling, lower limb swelling, respiratory distress, and spontaneous bleeding from the eyes, ears, nose, or tracheostomy site. Other findings included diarrhea, atrial fibrillation, and bilateral facial nerve paralysis. The overall mortality rate reported was 9% (6). Other reported complications of rattlesnake bites in horses include third-degree atrioventricular block, cardiac arrhythmias, cranial nerve deficits, laminitis, and congestive heart failure (7, 8).

The Mojave rattlesnake's toxin is described as either venom type A (Mojave Toxin), venom type B (proteolytic toxin), or a combination of venom type A and B. Venom A causes an ascending flaccid paralysis while venom B causes local tissue injury and hemorrhagic effects (9). It is speculated that the neurotoxicity associated with envenomation from non-Mojave rattlesnakes is due to variation in their venom causing more neurologic effects or due to the presence of Mojave toxin in the venom (5).

There are limited reports in the literature regarding neurotoxicity in domestic animals secondary to pit viper envenomation. There was a single case report where a dog developed respiratory paralysis after envenomation by a Southern Pacific Rattlesnake (9). Another retrospective study in small animals described the overall incidence of neurotoxicity from rattlesnake bites to be 5.4%. In this study, neurologic signs consisted of altered mentation, non-ambulatory tetraparesis or tetraplegia, extensor rigidity, conscious proprioceptive deficits, ambulatory tetraparesis, decreased spinal reflexes, ventral strabismus, muscle fasciculations, and seizures. Additionally respiratory paralysis was also noted in a subset of cases (5).

In humans, snakebite is a major health problem worldwide, with an estimated 5.4 million people bitten each year (10). Snake bites produce both local and systemic effects. The local effects include intense pain at the site of the bite, edema, ecchymosis, hyperemia, and inflammation (11).

Systemic effects include syncope, dyspnea, nausea, vomiting, and shock. Snake venom can impact the nervous system, leading to dizziness. Venom can affect the respiratory system, leading to shortness of breath. Cases with high toxicity and/or large volumes of venom can lead to shock. Hematologic disorders are usually characterized as mild but can lead to hemorrhages, which can be fatal. Furthermore, snake venom can activate the coagulation system, leading to DIC (11).

In one particular case, a 36-year-old maleIn one particular case, a 36-year-old male was bitten by an Artin viper (Vipera kaznakovi) on his lower extremity while collecting tea. The patient died the following day despite medical treatment. Autopsy revealed hemodynamic deterioration and multiple organ damage including changes in renal function. Ultimately, these complications from snake bite envenomation and anaphylaxis were determined as the cause of death in this case (12).

This is the first report of a *Crotalus oreganus* complex envenomation with neurologic signs in an equid. Based on the severe thrombocytopenia, marked facial swelling, and small patient size, the envenomation volume was likely severe. It is hypothesis-generating that the severity of envenomation in this equid resulted in the neurologic signs noted. Neurologic dysfunction should be monitored in equids post rattlesnake envenomation, particularly in smaller patients.

## Conclusion

This was the first report of urinary retention following rattlesnake envenomation in a horse. In this case, management with a urinary catheter and oral bethanechol was successful. No long-term complications were noted.

## Data Availability

The original contributions presented in the study are included in the article/supplementary material, further inquiries can be directed to the corresponding author.
